# A phase 1/2 study of chemosensitization with plerixafor plus G-CSF in relapsed or refractory acute myeloid leukemia

**DOI:** 10.1038/bcj.2017.21

**Published:** 2017-03-10

**Authors:** G L Uy, M P Rettig, R M Stone, M Y Konopleva, M Andreeff, K McFarland, W Shannon, T R Fletcher, T Reineck, W Eades, K Stockerl-Goldstein, C N Abboud, M A Jacoby, P Westervelt, J F DiPersio

**Affiliations:** 1 Division of Oncology, Department of Medicine, Washington University School of Medicine, St Louis, MO, USA; 2 Department of Medical Oncology, Dana-Farber Cancer Institute, Boston, MA, USA; 3 Department of Leukemia, The University of Texas MD Anderson Cancer Center, Houston, TX, USA; 4 Division of General Medical Sciences, Department of Medicine, Washington University School of Medicine, St Louis, MO, USA

The interaction of acute myeloid leukemia (AML) blasts with the bone marrow (BM) microenvironment provides potent protection against both spontaneous apoptosis and chemotherapy.^1^ Similar to normal hematopoietic stem cells (HSCs), AML blasts express many of the same adhesion molecules such as CXCR4, VLA-4, VLA-5 and CD44, which allow them to interact with the marrow microenvironment. We and others have demonstrated in murine models that CXCR4 inhibitors can mobilize AML cells from the BM into the peripheral blood and enhance the anti-leukemic effects of chemotherapy.^2, 3^ We have also demonstrated in a previous phase 1/2 study that plerixafor, a small molecule inhibitor of CXCR4, can be safely combined with chemotherapy in patients with relapsed or refractory AML with encouraging response rates.^4^

Mobilization of HSCs by granulocyte-colony stimulating factor (G-CSF) occurs through downregulation of mRNA and protein levels of CXCL12, the ligand for CXCR4.^5^ For mobilization of autologous HSCs, G-CSF acts synergistically when combined with plerixafor and mobilizes higher numbers of CD34^+^ cells compared with either agent alone.^6, 7^ In AML, ‘priming' with G-CSF concurrent with chemotherapy may result in superior outcomes for patients receiving induction therapy for AML.^8^ We hypothesized that disruption of the interaction between leukemic blasts with the marrow microenvironment using G-CSF in combination with plerixafor would effectively mobilize and sensitize AML blasts to chemotherapy.

In this phase 1/2 study (ClinicalTrials.gov NCT00906945), we evaluated the combination of G-CSF and plerixafor in conjunction with mitoxantrone, etoposide and cytarabine (MEC). Eligible participants were adults, age 18–70 years old, with relapsed or refractory AML. Subjects with a peripheral blood blast count of ⩾20 × 10^3^/mm^3^, acute promyelocytic leukemia, active central nervous system leukemia or who had been previously treated with MEC chemotherapy were excluded from the study. The primary endpoint in phase I was to determine the maximum tolerated dose and in phase II was to determine the complete response rate (CR+CRi) of plerixafor plus G-CSF in combination with MEC in patients with relapsed or refractory AML. The phase I was performed using a standard 3+3 design escalating to a maximum plerixafor dose of 0.75 mg/kg/day. For the phase II, a bivariate design was used in two stages to allow an interim analysis of toxicity and response rates.^9^ Treatment consisted of G-CSF 10 mcg/kg by subcutaneous injection daily on days 1–8. Plerixafor was administered intravenously (IV) on days 3–8. Chemotherapy consisting of mitoxantrone 8 mg/m^2^/day IV, etoposide 100 mg/m^2^/day IV and cytarabine 1000 mg/m^2^/day (MEC) was administered on days 4–8, ~4 h after administration of plerixafor.

Thirty-five patients with a median age of 56 years (range 29–70) were enrolled and treated on this study. The majority received treatment for first relapse (*n*=21, 60%) with 10 patients (29%) having had a prior allogeneic hematopoietic cell transplantation (Table 1). In the phase I, plerixafor was successfully escalated from 0.24 to 0.75^ ^mg/kg/day in five dose cohorts. The plerixafor 0.75 mg/kg/day dose was brought forward in the phase II expansion with a total of 20 patients treated in the first stage of the phase II. After an interim analysis, we observed that 6 out of 20 patients treated at the phase II dose achieved a CR/CRi (30%), which is less than the 7 out of 20 responses (35%) indicated in the study design for proceeding with the second stage of the phase II. As a result, the study was terminated for futility.

With a median follow-up of 34.6 months, the median overall survival for all subjects was 7.6 months with a 1-year overall survival of 37% (95% confidence interval: 21.2–53). The median time to neutrophil recovery (ANC⩾1000/mm^3^) was 40 days (range 23–62) from the start of treatment (36 days from MEC). The median time to platelet recovery (platelets ⩾100 K/mm^3^) was 32 days (range 30–62). Adverse events were typical of those observed for patients with relapsed or refractory AML with the most common non-hematologic adverse events of nausea (69%), vomiting (37%), febrile neutropenia (57%), headache (40%), fatigue (34%), fever (29%) and electrolyte abnormalities (hypocalcemia 37%, hypokalemia 31%) (Supplementary Table S1).

To determine the effect of G-CSF and plerixafor on leukemic cell mobilization, peripheral blood samples were collected at baseline, after G-CSF only on day 3 (pre-P), and at 2, 4, 6 and 24 h after IV plerixafor administration (Figure 1a). We were unable to detect a dose–response relationship between plerixafor and leukemic mobilization because of substantial interpatient variability in mobilization and the limited numbers of patients analyzed (Figures 1b and c). Peak mobilization of both total leukocytes and AML blasts occurred ~4–6 h after the administration of plerixafor on day 3 of G-CSF treatment and cell counts remained elevated at 24 h after administration (Figures 1b and c). In 31 evaluable patients, total leukocytes and blast cell counts increased a median of 2.4- and 3.5-fold, respectively, after 2 days of G-CSF (Figure 1d). Circulating levels of both leukocytes and blasts were further increased an additional 1.8- or 2.8-fold, respectively, at 6 h after plerixafor administration on day 3 (Figure 1d). The magnitude of AML blast cell mobilization was significantly greater than the total leukocyte mobilization at all treatment time points. Therefore similar to normal stem cell mobilization, plerixafor augments the mobilization of AML blasts by G-CSF.

We measured the expression of CXCR4 on AML blasts in response to administration of G-CSF and plerixafor using two different mAb clones. Plerixafor inhibits the binding of clone 12G5 to CXCR4. In contrast, the 1D9 mAb binds to the N-terminus of CXCR4 and is not affected by plerixafor. Similar to our previous trial,^4^ we observed a decrease in 12G5 binding on AML blasts from pretreatment to 6 h and an increase from 6 to 24 h toward baseline, indicating transient CXCR4 blockade by plerixafor *in vivo* (Figures 1e and f). In contrast, when CXCR4 was measured using 1D9, we found that there was an almost immediate (within 2 h) and dramatic increase of surface CXCR4 expression after plerixafor administration. Others and we have previously demonstrated that this upregulation of surface CXCR4 is primarily due to the plerixafor-mediated inhibition of CXCR4 internalization by its ligand CXCL12.^4, 10^

In addition to showing CXCR4 blockade by plerixafor *in vivo*, we also observed downregulation of the G-CSF receptor (CD114) and integrin alpha 6 (CD49f) on AML blasts by G-CSF (Figure 1f). In contrast, we observed no significant cell surface modulation of CD49d, CD62L, CD117 or CD135 on AML blasts following treatment with G-CSF and plerixafor (data not shown). However, we observed a slight but highly variable increase in the expression of the cell proliferation marker Ki67 in AML blasts following 2 days of G-CSF treatment (Figure 1f).

In this study, we sought to maximize blockage of the CXCL12/CXCR4 axis through (i) addition of G-CSF, which downregulates CXCL12 expression and acts synergistically with plerixafor in normal stem cell mobilization; (ii) intravenous instead of subcutaneous dosing of plerixafor to improve the kinetics of administration; and (iii) dose escalation of plerixafor to maximize CXCR4 blockade. Although we demonstrated the safety and feasibility of combining G-CSF and plerixafor with chemotherapy, combination therapy did not improve remission rates compared with historical controls.

We believe that primarily two factors contributed to the failure of G-CSF to enhance the efficacy of plerixafor in combination with MEC salvage chemotherapy in our clinical trial. First, a high proportion of patients with unfavorable prognosis were recruited to the study in comparison with our previous study, which was conducted at a single institution (Supplementary Table S2). Second, stimulation of cells with G-CSF has been shown to activate multiple signal transduction pathways that regulate the proliferation, differentiation and survival of myeloid cells. We believe that prosurvival signals mediated by G-CSF counteract both (i) the ‘priming' proliferative effect of G-CSF that increases the susceptibility of leukemic cells to cell cycle-specific chemotherapeutic agents and (ii) the pro-apoptotic effects of CXCR4 inhibition by plerixafor.

In our previous study of plerixafor with MEC, blast cell mobilization was not associated with remission.^4^ We hypothesize that inhibition of CXCR4-mediated prosurvival signaling is more important than the physical detachment and mobilization of AML blasts in enhancing the efficacy of MEC chemotherapy.

Plerixafor has a relatively short half-life of 4–5 h and is known to be a weak partial agonist of CXCR4.^11^ As high CXCR4 expression is a marker of poor prognosis in AML, plerixafor-mediated upregulation of CXCR4 may undermine the intended anti-apoptotic effect of CXCR4 blockade by also enhancing the re-homing of AML blasts to the BM, thus reducing the efficacy of CXCR4 inhibitor-based chemosensitization. A number of other CXCR4 inhibitors have been developed for clinical use including other small molecule inhibitors, antibodies and peptidomimetics. Compared with plerixafor, many of these agents provide more potent and sustained inhibition of CXCR4.^12, 13, 14^ These newer inhibitors also can directly induce apoptosis of AML and other tumor cells line *in vitro* which supports the assertion that antileukemic activity of CXCR4 inhibitors may be in part independent of the mobilization.^15^ We are currently exploring the use of these alternative CXCR4 inhibitors, as well as inhibitors of other pathways, which mediate tumor–stromal interactions in both AML and other hematologic malignancies.

## Figures and Tables

**Figure 1:**
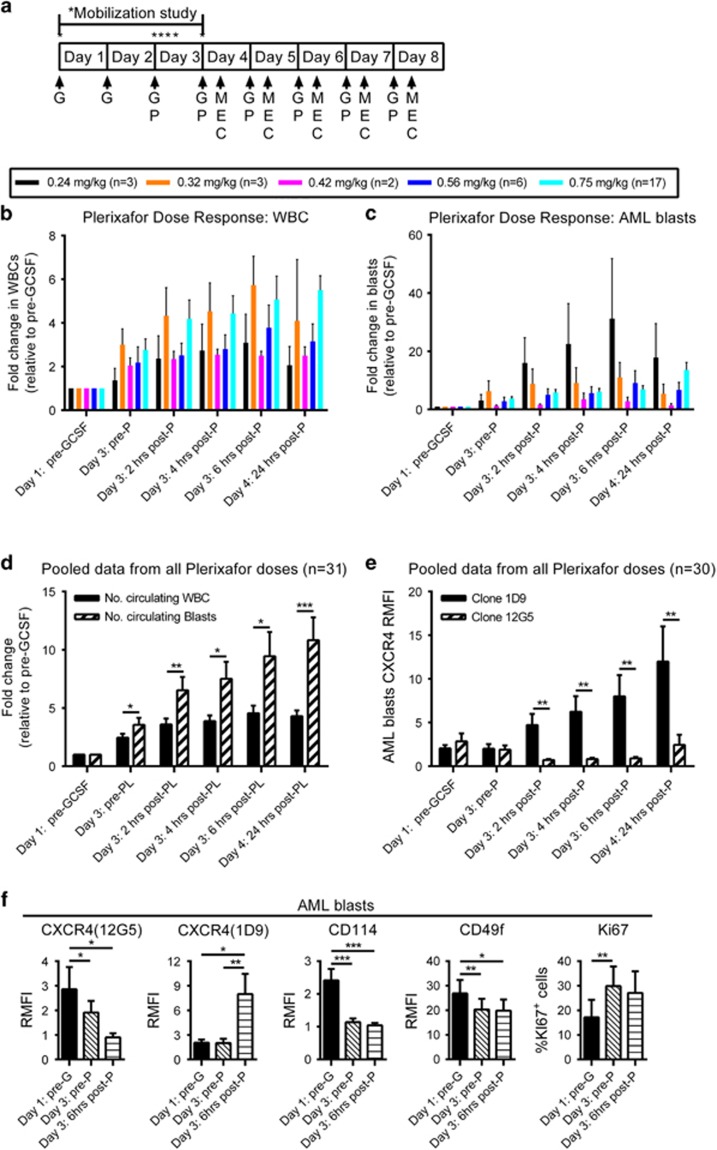
AML blast mobilization and phenotype. (**a**) Clinical trial schema. Patients with relapsed or refractory AML received G-CSF 10 mcg/kg daily for 8 days. Plerixafor (P) IV was administered on days 3–8. On days 4–8, Mitoxantrone 8 mg/m^2^/day, Etoposide 100 mg/m^2^/day and Cytarabine 1000 mg/m^2^/day (MEC) chemotherapy was administered 4 h after plerixafor. Peripheral blood samples were collected at baseline, after G-CSF only on day 3 (pre-P) and at 2, 4, 6 and 24 h after IV plerixafor administration. Complete blood counts including total leukocytes and blast (CD45^dim^/SSC^low^) counts were determined at each time point by flow cytometry. Mobilization of total CD45^+^ leukocytes (**b**) and CD45^dim^SSC^lo^ AML blasts (**c**) to the peripheral blood over time after administration of a single dose of plerixafor at 0.24, 0.32, 0.42, 0.56 or 0.75 mg/kg. Mean fold changes from baseline with SEM are shown. (**d**) Mobilization of total CD45^+^ leukocytes and CD45^dim^SSC^lo^ AML blasts to the peripheral blood over time from all evaluable patients enrolled in the trial. Mean fold changes from baseline with SEM are shown. Statistical comparisons were performed using a paired parametric Student's *t*-test. (**e**) The expression of CXCR4 on peripheral blood AML blasts was determined by flow cytometry using anti-CXCR4 monoclonal antibody clones 12G5 and 1D9. Mean fold changes in CXCR4 relative mean fluorescent intensity (RMFI) from baseline with SEM are shown. Statistical comparisons were performed using a paired parametric Student *t*-test. (**f**) Expression of clone 12G5 of CXCR4 (*n*=29), clone 1D9 of CD184 (*n*=29), CD114 (G-CSF receptor; *n*=17), CD49f (*n*=15) and Ki67 (*n*=10) on AML blasts. The RMFI or percentage of positive Ki67 cells with SEM are shown. Statistical comparisons were performed using a paired parametric Student's *t*-test. *P<0.05, **P<0.01 and ***P<0.001.

**Table 1:** Baseline patient characteristics (*n*=35)

*Patient characteristic*	
Age, median (range)	56 (29–70)
	
*Gender*	
Male (%)	18 (51)
Female (%)	17 (49)
	
*ECOG performance status*	
0 (%)	15 (43)
1 (%)	15 (43)
2 (%)	4 (11)
Missing (%)	1 (3)
	
*Onset of AML*	
*De novo* (%)	23 (66)
Therapy-related (%)	12 (34)
Prior MDS/MPN (%)	4 (11)
	
*Indication for treatment*	
Primary refractory (%)	10 (29)
First relapse (%)	21 (60)
Second relapse (%)	4 (11)
Prior unsuccessful salvage chemotherapy (%)	7 (20)
Prior allogeneic HCT (%)	10 (29)
	
*Cytogenetics*	
Favorable (%)	3 (9)
Intermediate (%)	19 (54)
Poor (%)	13 (37)
WBC k/cumm (range)	2.5 (0.2–14.7)
BM blast % (range)	33 (0–96)

Abbreviations: AML, acute myeloid leukemia; BM, bone marrow; ECOG, Eastern Cooperative Oncology Group; HCT, hematopoietic cell transplantation; MDS, myelodysplastic syndrome; MPN, myeloproliferative neoplasm; WBC, white blood cells.
