# Supplementary material for: A phase 1/2 study of chemosensitization with plerixafor plus G-CSF in relapsed or refractory acute myeloid leukemia
Source: Blood Cancer J. 2017 Mar 10;7(3):e542–. doi: 10.1038/bcj.2017.21 (PMC5380905; doi:10.1038/bcj.2017.21)
Supplement: Supplementary Information [file bcj201721x1.docx]

**Supplementary Information**

**A phase 1/2 study of chemosensitization with plerixafor plus G-CSF in relapsed or refractory acute myeloid leukemia**

**Trial design**

**This trial was a multicenter, open-label phase I/II study.** This study was registered at ClinicalTrials.gov with identifier NCT00906945 and was approved by the institutional review boards of Washington University School of Medicine, Dana-Farber Cancer Institute, and The University of Texas MD Anderson Cancer Center. The study was carried out in accordance with the Declaration of Helsinki and amendments. Written informed consent was obtained from all subjects prior to enrollment.

Patients were required to have an ECOG performance status ≤ 3, adequate organ function defined as calculated creatinine clearance ≥ 50 ml/min, AST, ALT, total bilirubin ≤ 2 x institutional upper limit of normal and left ventricular ejection fraction of ≥ 40%. The completed phase II portion includes a total of 46 patients, 20 in the first stage and 26 in the second stage. The minimum acceptable CR rate is 30%, and the expected CR rate with plerixafor is ≥ 50%. The maximum acceptable rate of prolonged cytopenias and regimen related mortality is 25%; the expected rate with plerixafor is ≤ 10%. The parameters for determining the stopping rules are as follows: probability of accepting a poor response (alpha 1) = 0.10, probability of accepting a toxic drug (alpha 2) = 0.15, and the probability of rejecting a good drug (beta) = 0.15 (i.e., 85% power to accept a good non-toxic treatment).

Responses to treatment were assessed according to the Revised Recommendations of the International Working Group for Diagnosis, Standardization of Response Criteria, Treatment Outcomes, and Reporting Standards for Therapeutic Trials in Acute Myeloid Leukemia.[^11^](#_ENREF_11) All patients who received at least one dose of plerixafor were considered evaluable for response. Adverse events were graded according to the Common Terminology Criteria for Adverse Events v4.0.

**Flow cytometry**

AML blasts were evaluated by flow cytometry using blood or BM samples collected in ethylenediaminetetraacetic acid (EDTA)-containing tubes and processed immediately. Samples were incubated for 30 min at 4°C with pre-titrated saturating dilutions of the following fluorochrome-labeled monoclonal antibodies (BD Biosciences, San Jose, CA; clone designated in parenthesis): CD3 (SK7), CD14 (MφP9), CD16 (3G8), CD19 (SJ25C1), CD33 (P67.6), CD34 (581), CD38 (HIT2), CD45 (Hi30), CD49d (9F10), CD49f (GoH3), CD62L (Dreg56), CD114 (LMM741), CD117 (YB5.B8), CD123 (7G3), CD184 (12G5 and 1D9) and KI67 (B56). Dead cells were excluded from these assays by staining with 2 μg/ml 7-amino-actinomycin D (Molecular Probes, Eugene, OR) for 5 min just prior to analysis. Appropriate isotype-matched negative controls were used to assess background fluorescence intensity. To obtain quantitative analysis of fluorescence intensity, histograms were made and the relative mean fluorescence intensity (RMFI) were calculated by dividing the mean fluorescence intensity of analyzed marker by the mean fluorescence intensity of respective isotype control**.** Absolute cell counts were performed using a lyse/no-wash protocol with SPHERO™ AccuCount fluorospheres (Spherotech Inc, Lake Forest, IL). Samples were analyzed on a Beckman Coulter Gallios flow cytometer or a BD Biosciences FACScan flow cytometer modified with a second, 633-nm laser and data were analyzed using FlowJo software (TreeStar, Ashland, OR). Statistical comparisons of flow cytometry data were performed using a paired parametric Student t-test (GraphPad Prism). P-values ≤0.05 were considered significant.

**Supplementary Table 1. Non-hematologic Adverse Events by CTCAE Grade and Count. Events exceeding 10% in total frequency are shown. (n=35)**

| **System Organ Class / Event Term** | **Grade 1** | **Grade 2** | **Grade 3** | **Grade 4** | **Grade 5** | **Total** | **(%)** |
| --- | --- | --- | --- | --- | --- | --- | --- |
| **Blood and lymphatic system disorders** |  |  |  |  |  |  |  |
| Anemia | 0 | 4 | 9 | 0 | 0 | 13 | (37) |
| Febrile neutropenia | 0 | 0 | 20 | 0 | 0 | 20 | (57) |
| **Gastrointestinal disorders** |  |  |  |  |  |  |  |
| Abdominal pain | 6 | 0 | 1 | 0 | 0 | 7 | (20) |
| Constipation | 5 | 2 | 0 | 0 | 0 | 7 | (20) |
| Diarrhea | 7 | 6 | 0 | 0 | 0 | 13 | (37) |
| Gastroesophageal reflux disease | 1 | 5 | 0 | 0 | 0 | 6 | (17) |
| Mucositis oral | 2 | 3 | 1 | 0 | 0 | 6 | (17) |
| Nausea | 21 | 3 | 0 | 0 | 0 | 24 | (69) |
| Vomiting | 12 | 0 | 1 | 0 | 0 | 13 | (37) |
| **General disorders and administration site conditions** | | |  |  |  |  |  |
| Chills | 5 | 0 | 0 | 0 | 0 | 5 | (14) |
| Edema limbs | 5 | 1 | 0 | 0 | 0 | 6 | (17) |
| Fatigue | 9 | 2 | 1 | 0 | 0 | 12 | (34) |
| Fever | 6 | 4 | 0 | 0 | 0 | 10 | (29) |
| Non-cardiac chest pain | 1 | 2 | 1 | 0 | 0 | 4 | (11) |
| Pain | 2 | 2 | 0 | 0 | 0 | 4 | (11) |
| **Infections and infestations** |  |  |  |  |  |  |  |
| Bacteremia | 0 | 0 | 4 | 0 | 0 | 4 | (11) |
| Lung infection | 0 | 1 | 5 | 0 | 2 | 8 | (23) |
| Sepsis | 0 | 0 | 0 | 4 | 4 | 8 | (23) |
| **Investigations** |  |  |  |  |  |  |  |
| Activated partial thromboplastin time prolonged | 5 | 0 | 0 | 0 | 0 | 5 | (14) |
| Alanine aminotransferase increased | 3 | 0 | 1 | 0 | 0 | 4 | (11) |
| Alkaline phosphatase increased | 3 | 1 | 0 | 0 | 0 | 4 | (11) |
| Aspartate aminotransferase increased | 4 | 0 | 0 | 0 | 0 | 4 | (11) |
| Blood bilirubin increased | 2 | 3 | 1 | 0 | 0 | 6 | (17) |
| INR increased | 7 | 0 | 0 | 0 | 0 | 7 | (20) |
| Neutrophil count decreased | 0 | 0 | 0 | 7 | 0 | 7 | (20) |
| Platelet count decreased | 0 | 0 | 0 | 15 | 0 | 15 | (43) |
| White blood cell decreased | 0 | 0 | 0 | 16 | 0 | 16 | (46) |
| **Metabolism and nutrition disorders** |  |  |  |  |  |  |  |
| Anorexia | 4 | 2 | 0 | 0 | 0 | 6 | (17) |
| Hypoalbuminemia | 3 | 6 | 0 | 0 | 0 | 9 | (26) |
| Hypocalcemia | 2 | 11 | 0 | 0 | 0 | 13 | (37) |
| Hypokalemia | 5 | 2 | 2 | 2 | 0 | 11 | (31) |
| Hypomagnesemia | 7 | 0 | 0 | 0 | 0 | 7 | (20) |
| Hyponatremia | 4 | 0 | 1 | 0 | 0 | 5 | (14) |
| **Musculoskeletal and connective tissue disorders** | | |  |  |  |  |  |
| Bone pain | 4 | 2 | 2 | 0 | 0 | 8 | (23) |
| **Nervous system disorders** |  |  |  |  |  |  |  |
| Dizziness | 4 | 1 | 0 | 0 | 0 | 5 | (14) |
| Headache | 7 | 6 | 1 | 0 | 0 | 14 | (40) |
| Paresthesia | 4 | 1 | 0 | 0 | 0 | 5 | (14) |
| **Psychiatric disorders** |  |  |  |  |  |  |  |
| Insomnia | 5 | 2 | 0 | 0 | 0 | 7 | (20) |
| **Renal and urinary disorders** |  |  |  |  |  |  |  |
| Proteinuria | 3 | 1 | 0 | 0 | 0 | 4 | (11) |
| **Respiratory - thoracic and mediastinal disorders** | |  |  |  |  |  |  |
| Cough | 3 | 2 | 0 | 0 | 0 | 5 | (14) |
| Dyspnea | 3 | 3 | 0 | 0 | 0 | 6 | (17) |
| Pneumonitis | 1 | 3 | 0 | 0 | 1 | 5 | (14) |
| **Skin and subcutaneous tissue disorders** |  |  |  |  |  |  |  |
| Rash maculo-papular | 3 | 3 | 0 | 0 | 0 | 6 | (17) |
| **Vascular disorders** |  |  |  |  |  |  |  |
| Hypotension | 4 | 4 | 2 | 0 | 0 | 10 | (29) |

**Response rate by treatment group and site**

Several studies have examined baseline patient characteristics which predict response to salvage chemotherapy in AML.^2, 3^ Specifically, the duration of the initial CR and prior number of salvage chemotherapy regimens are highly predictive of response to subsequent therapy.^4^ After reviewing patient characteristics and responses from each of the 3 study sites, we observed an imbalance in the characteristics of patients recruited at a single center which likely contributed to the lower than expected response rate (**Supplementary Table 2**).

| **Supplementary Table 2. Predicted versus observed response rate by treatment group and site** | | | | | | |
| --- | --- | --- | --- | --- | --- | --- |
|  |  |  |  |  |  |  |
| **Response** | **Group 1 (n=4)** | **Group 2 (n=4)** | **Group 3 (n=20)** | **Group 4 (n=7)** |  |  |
| Predicted CR/CRi % | 73% | 47% | 14% | 0% |  |  |
| Observed CR/CRi % | 75% (n=3) | 50% (n=2) | 15% (n=3) | 14% (n=1) |  |  |
|  |  |  |  |  |  |  |
| **Site** | **Group 1 (n=4)** | **Group 2 (n=4)** | **Group 3 (n=20)** | **Group 4 (n=7)** | **Predicted CR** | **Observed CR** |
| Site #1 (n=21) | 3 | 3 | 13 | 2 | 26% | 33% |
| Site #2 (n=6) | 1 | 1 | 4 | 0 | 29% | 33% |
| Site #3 (n=8) | 0 | 0 | 3 | 5 | 5% | 0 |
| Group 1: initial CR duration > 2 yrs receiving their 1st salvage | | | |  |  |  |
| Group 2: CR 1-2 yrs, receiving 1st salvage | | |  |  |  |  |
| Group 3: CR < 1 yr, no initial CR, 1st salvage | | |  |  |  |  |
| Group 4: CR < 1 yr, no initial CR, 2nd or subsequent salvage attempt | | | | |  |  |

**REFERENCES**

1. Uy GL, Rettig MP, Motabi IH, McFarland K, Trinkaus KM, Hladnik LM*, et al.* A phase 1/2 study of chemosensitization with the CXCR4 antagonist plerixafor in relapsed or refractory acute myeloid leukemia. *Blood* 2012 Apr 26; **119**(17)**:** 3917-3924.

2. Breems DA, Van Putten WL, Huijgens PC, Ossenkoppele GJ, Verhoef GE, Verdonck LF*, et al.* Prognostic index for adult patients with acute myeloid leukemia in first relapse. *J Clin Oncol* 2005 Mar 20; **23**(9)**:** 1969-1978.

3. Keating MJ, Kantarjian H, Smith TL, Estey E, Walters R, Andersson B*, et al.* Response to salvage therapy and survival after relapse in acute myelogenous leukemia. *J Clin Oncol* 1989 Aug; **7**(8)**:** 1071-1080.

4. Estey E, Kornblau S, Pierce S, Kantarjian H, Beran M, Keating M. A stratification system for evaluating and selecting therapies in patients with relapsed or primary refractory acute myelogenous leukemia. *Blood* 1996 Jul 15; **88**(2)**:** 756.
